# Heat/mortality sensitivities in Los Angeles during winter: a unique phenomenon in the United States

**DOI:** 10.1186/s12940-018-0389-7

**Published:** 2018-05-03

**Authors:** Adam J. Kalkstein, Laurence S. Kalkstein, Jennifer K. Vanos, David P. Eisenman, P. Grady Dixon

**Affiliations:** 1 Department of Geography and Environmental Engineering, Center for Languages, Cultures, and Regional Studies, United States Military Academy, 745 Brewerton Rd; 6th Floor, West Point, NY 10996 USA; 2 Department of Public Health Sciences, Miller School of Medicine, University of Miami, Miami, FL USA; 3 Scripps Institution of Oceanography & School of Medicine, University of California San Diego, La Jolla, CA USA; 4 UCLA Center for Public Health and Disasters, David Geffen School of Medicine at UCLA, Los Angeles, CA USA; 5 Department of Geosciences, Fort Hays State University, Hays, KS USA

**Keywords:** Heat, Human mortality, Winter heat waves, Los Angeles, Air masses, DLNM

## Abstract

**Background:**

Extreme heat is often associated with elevated levels of human mortality, particularly across the mid-latitudes. Los Angeles, CA exhibits a unique, highly variable winter climate, with brief periods of intense heat caused by downsloping winds commonly known as Santa Ana winds. The goal is to determine if Los Angeles County is susceptible to heat-related mortality during the winter season. This is the first study to specifically evaluate heat-related mortality during the winter for a U.S. city.

**Methods:**

Utilizing the Spatial Synoptic Classification system in Los Angeles County from 1979 through 2010, we first relate daily human mortality to synoptic air mass type during the winter season (December, January, February) using Welch’s t-tests. However, this methodology is only somewhat effective at controlling for important inter- and intra-annual trends in human mortality unrelated to heat such as influenza outbreaks. As a result, we use distributed lag nonlinear modeling (DLNM) to evaluate if the relative risk of human mortality increases during higher temperatures in Los Angeles, as the DLNM is more effective at controlling for variability at multiple temporal scales within the human mortality dataset.

**Results:**

Significantly higher human mortality is uncovered in winter when dry tropical air is present in Los Angeles, particularly among those 65 years and older (*p* < 0.001). The DLNM reveals the relative risk of human mortality increases when above average temperatures are present. Results are especially pronounced for maximum and mean temperatures, along with total mortality and those 65 + .

**Conclusions:**

The discovery of heat-related mortality in winter is a unique finding in the United States, and we recommend stakeholders consider warning and intervention techniques to mitigate the role of winter heat on human health in the County.

## Background

The negative impact of heat on human health is well-established, and extreme heat is often associated with elevated levels of human mortality, particularly across the mid-latitudes [1–4]. Despite its seemingly moderate Mediterranean climate, previous research has demonstrated that heat-related illness is considerable across Los Angeles County, California, largely because of the highly variable summer climate in the area [5]. During summer, there are long stretches of benign weather, but these are punctuated by periods of intense heat often attributed to Santa Ana wind situations, when temperatures can reach 37 °C or more, even close to the coast. Such rapid variation in weather, even more so than the intensity of the heat itself, is responsible for short-term mortality increases in cities around the United States; thus, most heat-related deaths are concentrated in the northeastern and midwestern part of the country [6], but also along the Pacific Coast from southern California up to Seattle [7, 8]. Many of these deaths are preventable if proper warnings are given to the general population, and if effective intervention activities are put into place by stakeholders in major urban areas [7, 9].

The unique, highly variable climate across Los Angeles County also extends into the winter season, and previous research has demonstrated that larger winter-season temperature variability is associated with increases in human mortality across a variety of climate types around the world [10, 11]. In Los Angeles, the Santa Ana winds, which are most prevalent in the winter, bring brief periods of intense dry heat, and occur when surface high pressure is situated over the Great Basin or Rocky Mountains with lower pressure off the southern California coast [12]. The resulting pressure gradient produces a northeast or east wind, transporting an air mass from the Mojave Desert into the Los Angeles Metropolitan Area. An overall decrease in elevation from east to west warms the air adiabatically, producing even hotter, drier conditions.

It is plausible that these oppressive thermal conditions in the winter can be detrimental to human health, particularly since they are most likely to occur at the time of year when the local population would be least acclimatized to intense heat, and thus, most susceptible to adverse heat-health effects [13, 14]. In fact, there are winter days in which Los Angeles has exhibited the highest temperatures of any major city in the U.S., including Phoenix, AZ [15]. Interestingly, temperature-mortality relationships in Barcelona, Spain, which has a somewhat similar climate to Los Angeles, were stronger in the winter than summer [16].

The goal of this research is to determine if Los Angeles County is susceptible to heat-related mortality during the winter season, when rare but intense heat episodes occur. More specifically, we relate daily human mortality across Los Angeles County to both synoptic air mass type and thermal situations to determine if oppressive atmospheric conditions result in elevated levels or heightened risk of human mortality. To our knowledge, this is the first study in the U.S. to examine heat-related mortality solely in the winter when most mid-latitude cities do not experience temperatures high enough to negatively impact human health. This study was conducted as part of a larger heat mortality study commissioned by the Los Angeles County Department of Public Health, and results are intended to inform heath emergency adaptation planners for the department.

## Methods

### Meteorological data

Weather data are supplied by the National Centers for Environmental Information (NCEI) and include daily maximum, minimum, and average temperatures [17]. Each day was also classified into an air mass category for Los Angeles using the Spatial Synoptic Classification (SSC), which requires four-times daily meteorological data including temperature, dew point, cloud cover, and pressure [18]. The SSC, which has been used extensively in climate/human health analyses [19, 20], places each day into one of a number of air mass types listed in Table 1. An air mass is defined as a body of air that is relatively homogeneous in terms of temperature, atmospheric moisture, and other meteorological characteristics along its horizontal extent [21], and previous research suggests that humans respond to the simultaneous effects of a large number of meteorological elements, rather than just individual weather variables [22]. As air masses represent entire “weather situations” rather than only individual weather elements, they present a more comprehensive determination of the atmospheric environment.

**Table 1:** Air mass types in the SSC

Air Mass	Definition
Generally Non-Oppressive Air Masses	
Dry Polar (DP)	Arrives from polar regions and is usually associated with the lowest temperatures observed in a region for a particular time of year as well as clear, dry conditions.
Dry Moderate (DM)	Consists of mild and dry air. Often occurs when air warms as it descends mountain ranges.
Moist Polar (MP)	Typically cloudy, humid, and cool. MP air appears when air over the adjacent cool ocean is brought inland, frequently during stormy conditions.
Moist Moderate (MM)	Considerably warmer and more humid than MP. The MM air mass typically appears in a zone south of MP air, near an adjacent stationary front (an area where warm air moves over a cooler air mass).
Moist Tropical (MT)	Warm and very humid. It is typically found in warm sectors of mid-latitude cyclones or in a return flow on the western side of a high-pressure area.
Transition (TR)	Defined as days in which one weather type yields to another, based on large shifts in pressure, dew point, and wind over the course of the day.
Oppressive Hot Air Masses	
Dry Tropical (DT)	Represents the hottest and driest conditions found at any location. There are two primary sources of DT: either it is transported from the desert regions, such as the Sonoran Desert, or it is produced by rapidly descending air.
Moist Tropical Plus (MT+)	Hotter and more humid subset of MT. It is defined as an MT day where both morning and afternoon temperatures are above the MT averages, and thus captures the most “oppressive” subset of MT days.

The SSC is the basis of numerous “heat-health warning systems”, which are currently in use by U.S. National Oceanic and Atmospheric Administration/National Weather Service Offices (NWS) and similar entities around the world to call excessive heat warnings [23–25]. In addition, the procedure has been utilized in a number of climate change-health analyses [5, 8, 26]. Thus, the air mass-based approach is well-suited to examine the potential impact of heat on human health across Los Angeles County. Here, we are most interested in the two “oppressive” air masses, which have historically been associated with statistically significantly higher daily mortality when they are present over a region: dry tropical (DT) and moist tropical plus (MT+) [5, 27].

The use of the appropriate meteorological station(s) in this analysis is something that must be given serious consideration. First, there are only three stations that provide us with the hourly data necessary to develop the SSC: Los Angeles International Airport (LAX), Burbank Bob Hope Airport (BUR), and the Marine Corps Air Station in El Toro (NZJ). All other stations around Los Angeles County either possess too short a record to be of use, or are of the “cooperative” type, which only measure maximum and minimum temperatures and total precipitation for the day. These are inadequate to develop the detailed air mass analyses that are required for the SSC. Ultimately, we decided against using LAX as it is influenced by the cooler ocean due to its proximity to the coast, and thus does not represent what populations in the interior of Los Angeles would experience. In addition, the weather record at NZJ was too short (1989–1997). These issues resulted in BUR as a natural choice for the meteorological data and air mass classification, and its proximity to some lower-income and high population density areas in Los Angeles County is also beneficial.

Although individual weather variables may vary across Los Angeles County, the *weather situation* is almost always the same throughout the County, with the possible exceptions being the immediate coast and if a cold front or some other macro-scale feature is apparent within the area. This is partially what makes the SSC unique: the quantification of the *weather situation*, or air mass, that is apparent over the region. It is possible that, at high elevations, a particular air mass may be so physically different that it has a dissimilar impact on human well-being. But considering that most of the population within Los Angeles County does not live in in unique regions such as Angeles National Forest, we feel comfortable with the use of single, detailed weather situations for this study, similar to other cities that have been analyzed in past heat-health studies.

Considering that the primary goal of this manuscript is to examine heat-related mortality throughout the winter, we only examine the meteorological winter season defined as December, January, and February spanning from 1979 through 2010, years in which complete human mortality data and meteorological data are available.

### Mortality data

We obtained daily mortality data across Los Angeles County for December, January, and February, 1979 through 2010, from the Centers for Disease Control and Prevention, National Vital Statistics System [28]. We did not examine heat-related mortality based on various International Classification of Diseases (ICD) code groups, partially because the use of any “heat-related mortality” designations (ICD-10 designations of T67.x) has been shown to grossly underestimate the number of heat deaths, many of which are only found by evaluating “spikes” in mortality during hot weather events [29, 30]. Thus, all-cause mortality was used here, and it has been shown to be among the most robust estimators of determining mortality spikes associated with intense heat events [24, 31]. In addition to total daily mortality counts, we also examined two distinct age groups: those below 65 and those 65 and above.

An important step in many heat-health studies is to standardize the mortality data to eliminate as much non-environmental “noise” as possible. This includes adjusting for natural seasonal cycles in human mortality, along with any inter- or intra-annual changes in population. To accomplish this goal, and remaining consistent with the methodology employed by other heat-health studies [25, 32], we fitted a polynomial function for average daily mortality for the winter season across the years of record, representing baseline deaths for each year. Daily mortality was then expressed as a deviation from this baseline. However, there is also a within-season pattern associated with winter mortality, and average daily values across Los Angeles peak in early January. Using a 15-day running mean on day-of-year mortality averages throughout the winter season, we adjusted for this pattern. The result is a daily mortality value expressed as departure from average, which in theory, controls for both long-term and seasonal trends in mortality. Using Welch’s t-tests, these mortality data were used to compare daily SSC air mass type to mortality anomalies to determine if any specific air mass type is associated with statistically significant increases in human mortality.

**Fig. 1:**
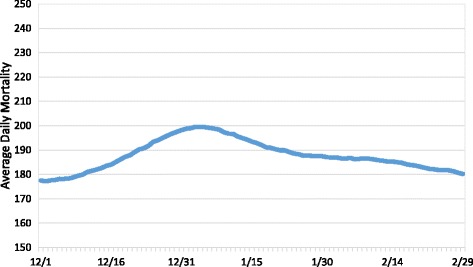
Average daily mortality for Los Angeles County from 1979 through 2010. A 15-day running mean was used to smooth the data

**Fig. 2:**
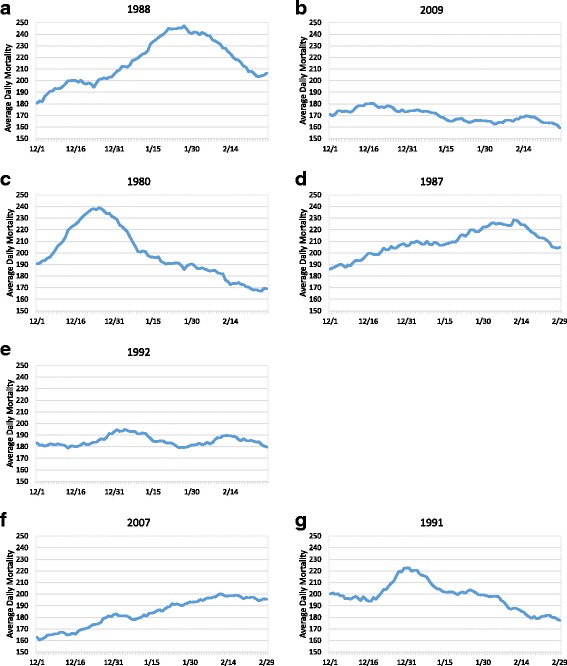
Average daily mortality for Los Angeles County for the winters of 1988/1989 (**a**), 2009/2010 (**b**), 1980/1981 (**c**), 1987/1988 (**d**), 1992/1993 (**e**), 2007/2008 (**f**), and 1991/1992 (**g**). 15-day running means were used to smooth the data

Although the above methodology has been effective at examining heat-related mortality during the summer, it became evident that it was less effective for the winter. With the notable exception of isolated heat events, summer mortality in Los Angeles County tends to be relatively stable, with similar values from year-to-year. However, winter mortality values can vary wildly, both in timing and magnitude, primarily as a result of the presence and severity of influenza outbreaks [33, 34]. Over the December through February, 1979–2010 period of record, average daily mortality in Los Angeles peaked in early January with just over 200 deaths (Fig. 1). However, when similar mortality trends are plotted for each year (we use a 15-day running mean to smooth the data), the patterns vary dramatically. Some years experience high overall mortality, others low (Fig. 2a, b). Some years have peak mortality early in the winter season (Fig. 2c), others peak late (Fig. 2d), while a few years exhibit two distinct peaks (Fig. 2e). In several cases, mortality increases throughout the winter season (Fig. 2f), while other years experience a steady decrease (Fig. 2g).

Considering the varying trends in winter mortality are likely due to influenza, which is largely unaffected by short-term heat waves [35], we attempted to remove the flu signal by examining only mortality *not* attributed to respiratory distress (ICD-10 J00-J99). However, this methodology also proved ineffective since influenza tends to increase other causes of death beyond respiratory distress, including heart attack and stroke [36]. Thus, yet another alternate methodology was needed.

### Distributed lag nonlinear model (DLNM)

After initial analyses of the observational data provided some insight into the temperature-mortality associations, we used the DLNM package in R (http://cran.r-project.org/web/packages/dlnm/) to refine the comparisons using all available temperature data rather than focusing on discrete heat events or specific air mass types. The DLNM package offers the ability to control for variability at multiple scales, which makes it the best choice for analyzing inconsistent variations like those that are apparent in winter mortality for Los Angeles County.

We applied the general linear model regression function to use daily temperature values (maximum, mean, and minimum) to create estimations of raw daily mortality counts. Natural cubic splines allow for normalization with respect to various time scales. We compiled the model for the winter months (December, January, February) with two equally spaced spline knots and 4° of freedom consistent with previous research [37, 38]. We also employed a categorical “day of week” spline to account for weekly cycles/patterns [39]. Initial models were run with 10-day lags, and 3-D plots of relative risk variation with temperature and lag were used to determine the appropriate lags for remaining models. The relative risk of mortality (RR) due to the various temperature exposures was compared to the location’s median temperature value for the winter months during the study period.

## Results

Los Angeles displays a generally moderate climate with an average daily maximum temperature across the period of record of 20.2 °C and an average daily minimum of 7.7 °C (Table 2). The two historically oppressive air mass types, DT and MT+ are the two hottest and occur on 19.1 and 1.8% of days, respectfully. While DT has higher daily maximum temperatures, both minimum and average temperatures are higher within MT+.

**Table 2:** Average daily meteorological conditions at Burbank Bob Hope Airport for each air mass type for the winter season (December, January, and February) from 1979 to 2010

Air mass	Air mass frequency (%)	Average maximum temperature (°C)	Average minimum temperature (°C)	Average daily temperature (°C)
Dry Moderate	33.0	19.3 (3.1)	5.5 (2.0)	12.4 (2.2)
Dry Polar	1.9	14.8 (3.0)	3.5 (2.8)	9.2 (2.3)
**Dry Tropical**	**19.1**	**25.7 (2.8)**	**9.2 (2.8)**	**17.4 (2.3)**
Moist Moderate	13.8	16.8 (2.4)	9.9 (2.0)	13.3 (1.7)
Moist Polar	5.6	14.5 (2.4)	7.1 (2.1)	10.8 (1.8)
Moist Tropical	8.9	21.2 (2.5)	10.1 (1.9)	15.7 (1.6)
Transition	10.4	20.0 (4.6)	7.2 (3.3)	13.6 (3.3)
**Moist Tropical Plus**	**1.8**	**24.2 (3.0)**	**11.8 (1.8)**	**18.0 (1.6)**
Missing	5.5	n/a	n/a	n/a
Total	100	20.2 (4.5)	7.7 (3.2)	14.0 (3.1)

Standard deviations are in parentheses, and oppressive air masses are in bold

**Fig. 3:**
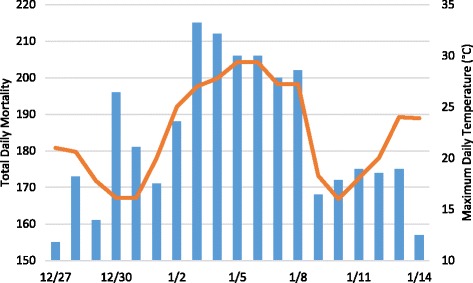
Total daily mortality in Los Angeles County (bars) and maximum daily temperature (line) from 27 December, 2002 through 14 January, 2003

While a cursory, visual examination of extreme heat events illustrates that severe heat is frequently associated with large, immediate increases in human mortality (Fig. 3), Welch’s t-tests showed that DT air is associated with an average of 4.4 total excess deaths per day with 3.7 excess deaths among those 65 years and older (Table 3). Both values are statistically significant (*p* < 0.001). The only other statistically significant values (*p* < 0.01) are associated with decreases in average mortality unrelated to heat-related illness. Mortality during DT air masses is also the most variable and displays the largest standard deviations for both total mortality and the 65+ age segment.

**Table 3:** Average daily mortality (expressed as departure from average) within each air mass type for each age group

Air Mass	Average Daily Mortality (Total)	Average Daily Mortality (< 65)	Average Daily Mortality (> = 65)
Dry Moderate	−0.7 (20.3)	− 0.2 (9.0)	− 0.5 (16.8)
Dry Polar	2.9 (21.2)	2.7 (9.0)	0.3 (17.8)
Dry Tropical	**4.4 (22.7)**	0.8 (8.5)	**3.7 (19.4)**
Moist Moderate	**−2.8 (20.0)**	−0.7 (8.5)	− 2.1 (17.0)
Moist Polar	1.3 (19.7)	0.6 (8.7)	0.8 (16.8)
Moist Tropical	− 1.7 (17.5)	−0.9 (8.9)	−0.8 (14.5)
Transition	−0.1 (19.7)	1 (9.0)	−1.1 (15.8)
Moist Tropical Plus	0.0 (18.6)	−2.3 (10.4)	2.3 (16.0)
Missing	**−4.5 (18.9)**	−1.1 (8.2)	**−3.5 (16.0)**

Standard deviations are in parentheses, and statistically significant values are in bold (*p* < 0.01)

**Fig. 4:**
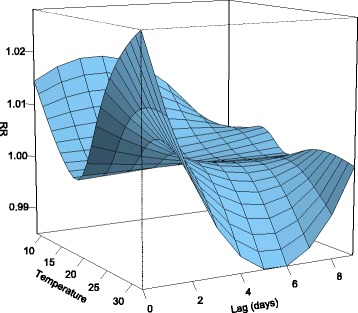
3-D plot of relative risk variation in mortality for those 65+ with daily maximum temperature at various lag times

A 3-D plot of relative risk variation in mortality for those 65+ with daily maximum temperature at various lag times shows that anomalously warm days are associated with immediate responses in mortality and that the response is greater than any associated with cooler days throughout the 10-day period (Fig. 4). As pointed out by Gasparrini [40], these tri-dimensional plots are valuable as summaries of the statistical associations, but they cannot be relied upon for specific inferences [40]. Accordingly, we are less concerned with the RR values from Fig. 4, as those values are provided by later results. The purpose of 3-D plot is to justify our chosen lag of 0 days (i.e., mortality events associated with temperature on the same day).

**Fig. 5:**
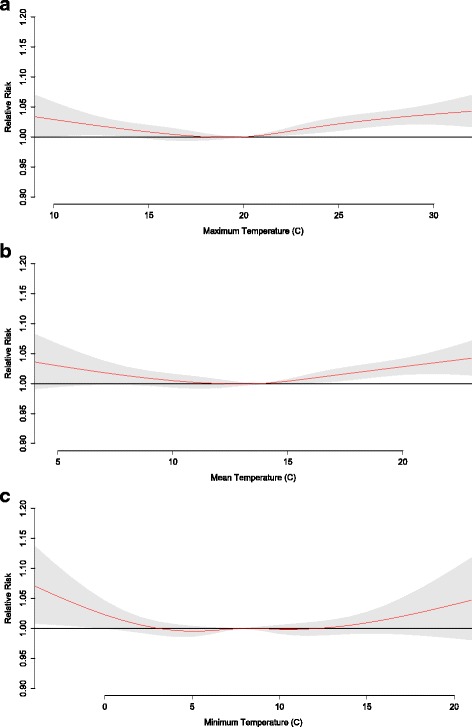
Relative risk for mortality of all ages at various daily (**a**) maximum, (**b**) mean, and (**c**) minimum temperatures. The red line is the predicted value and the gray shading represents the 95% confidence interval

**Fig. 6:**
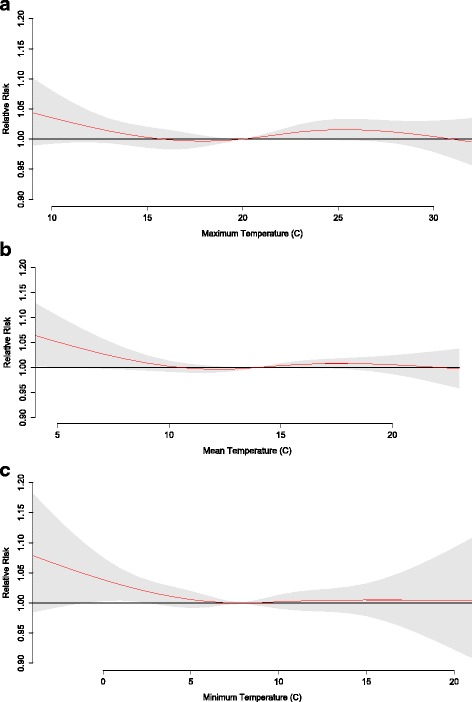
Relative risk for mortality of ages < 65 at various daily (**a**) maximum, (**b**) mean, and (**c**) minimum temperatures. The red line is the predicted value and the gray shading represents the 95% confidence interval

**Fig. 7:**
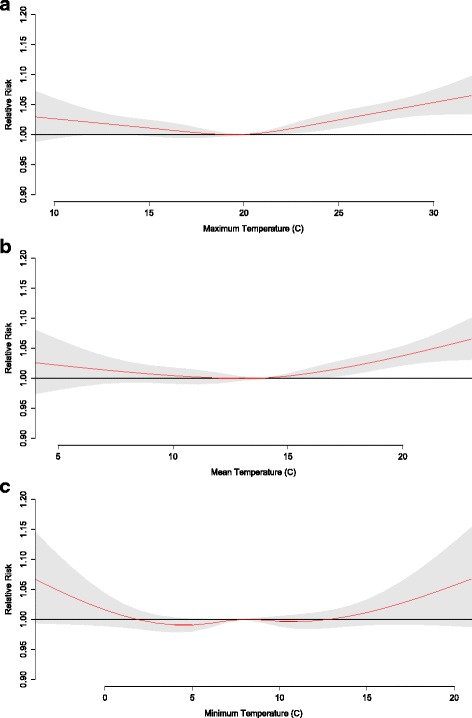
Relative risk for mortality of ages 65+ at various daily (**a**) maximum, (**b**) mean, and (**c**) minimum temperatures. The red line is the predicted value and the gray shading represents the 95% confidence interval

The RR of mortality at various temperature exposures shows that risk generally increases with both anomalously high and low temperatures, although RR is generally more impacted by excessive heat than excessive cold as indicated by significant effect estimates under higher temperatures (Figs. 5, 6 and 7). The risk of mortality due to temperature is most robust among total mortality and those 65+, with significant effect estimates for maximum temperatures of 12 °C above average for the season (RR = 1.04 (CI 1.02–1.07), RR = 1.07 (CI 1.03–1.09), respectively) and mean temperatures of 9 °C above average for the season (RR = 1.04 (CI 1.01–1.07), RR = 1.07 (CI 1.03–1.10), respectively). Temperature-mortality RR is generally less evident for the < 65 age segment and for minimum temperatures (no significant RR estimates).

## Discussion

It is apparent that winter heat can have a negative impact on human health in Los Angeles County, as demonstrated using two approaches in the current manuscript. This winter heat-mortality relationship is an unusual finding among U.S. cities and is an important discovery, particularly since the Los Angeles Department of Public Health and other responding agencies in Los Angeles, including the NWS, were not aware of such an impact. Further, the impact of extreme winter heat on human health is substantial. Excess human mortality is apparent within DT air masses, a synoptic situation that occurs on 19.1% of winter days in Los Angeles County. Likewise, relative risk for human mortality increases significantly with anomalously high winter temperatures. Considering the large population across Los Angeles County, these results suggest that a substantial loss of life can occur during oppressively hot winter conditions.

It is not surprising that the elderly (those 65 years and older) are disproportionately affected by extreme heat. In fact, both the synoptic (air mass) approach and DLNM methodologies display strong heat-health relationships within this age segment. This large response is consistent with summer excessive heat research, and the elderly have consistently been found to be more susceptible to extreme heat as a result of a decreased ability of the body to cope with oppressive conditions [41–43]. However, this effect has not been well-documented for winter excessive heat.

Why is the Los Angeles urban area so susceptible to winter heat and associated negative health outcomes? One reason is the unique nature of Los Angeles’ winter weather. There is no large city in the U.S. that exhibits such excessive heat in the winter as Los Angeles. A comparison between daily high record temperatures in Los Angeles as compared to Phoenix during the three winter months of December, January, and February shows that when it is hot in winter, Los Angeles easily exceeds Phoenix in maximum temperature. Both cities have detailed meteorological records from areas near downtown for at least 120 years, and when comparing daily all-time records, Phoenix doesn’t even approach Los Angeles when it comes to winter heat. Virtually every day in Los Angeles during the 3 month winter period exhibits a historical maximum daily temperature record equaling or exceeding 85 °F (29.4 °C), while in Phoenix, very few historical maximum daily temperature records exceed this threshold. The differentials between the two cities are equally dramatic when comparing maximum daily records equaling or exceeding 90 °F (32.2 °C). Except for late February, when solar angles are increasing, there are no winter maximum daily temperature records equaling or exceeding 90 °F in Phoenix. In Los Angeles, winter maximum daily temperature records can exceed 90 °F, and the monthly maximum temperature records for the city in January and February are an astonishing 95 °F (35 °C). Local Santa Ana modification of DT air allows for these excessive temperatures to be reached, and temperatures above 90 °F occur, on average, every few years in Los Angeles, but usually in consecutive day strings during an excessive heat event. This is unprecedented for any city in the U.S., including winter-warm areas like South Florida and the Desert Southwest. Also of interest is the lack of significant mortality response for MT+ air masses, which are often found to be “offensive” along with the DT air mass in summer in Los Angeles and other cities [44]. It is clear that MT+ does not reach high enough temperatures in the winter to create widespread health problems, while DT occasionally does, particularly with the higher solar loads associated with DT’s transparent atmosphere.

The urban structure of the Los Angeles Basin is ill-equipped to handle hot weather situations, which may contribute to the sensitivity of Los Angeles residents to negative heat/health outcomes in both summer and winter [45]. Unlike Phoenix and Miami, where housing and general architecture are more amenable to hot weather conditions, Los Angeles’ housing composition is generally less adapted, particularly if home air conditioning is unavailable [46]. In Los Angeles County, fewer than 50% of households possess central air conditioning; no doubt others have window units, but the proportion of homes with air conditioning is considerably lower in Los Angeles than in other cities, including Phoenix and most eastern large cities [45].

Another possible explanation to the observed winter sensitivity is the “surprise element” of excessive winter heat. Los Angeles residents are not warned about the onset of extremely hot weather in winter in a manner similar to the warnings issued by the NWS and allied stakeholders in summer. Stakeholders are not prepared for heat-related illnesses in winter as they are in summer, nor are most even aware that there is a winter heat-health problem. Thus, there seems to be a “stealthy” aspect to the winter heat phenomenon, which probably plays a role in exacerbating the surprisingly large negative health impact.

An unexpected finding is the large year-to-year variation in both the magnitude and timing of winter all-cause mortality in general (Fig. 2). These patterns, which are largely caused by the timing and magnitude of infectious disease outbreaks, limit the effectiveness of common standardization techniques that use a best-fit linear or polynomial algorithm to control for typical seasonal patterns in mortality. These year-to-year variations are a vital component for researchers to consider when conducting human mortality research throughout the winter influenza season. We strongly recommend that future winter mortality studies take this year-to-year variability into account rather than assuming a similar winter pattern for all years, which is what is often done for summer studies of this type.

It remains unclear why some days seem particularly severe in their impact while others are more benign, as demonstrated by high standard deviations in human mortality when DT air is present. One possible explanation is the natural variability in human mortality. Since heat is not the only factor to impact human health, other causes of variation may be at play here (e.g. holidays, large outdoor events, other environmental factors), resulting in some DT days with only a small change in human mortality, some days with large spikes in mortality, and most days with a moderate increase in mortality. Still, future work is necessary to help isolate any environmental factors that might lead to increased likelihoods of elevated mortality, particularly if the human health outcomes of extreme heat are to be forecast in advance.

Although air pollution is often associated with heightened levels of mortality during the presence of hot air masses [47–51] in Los Angeles, this relationship is likely only present during summer and spring heat events. Using the SSC and ground-based stations, Liu et al. [52] found that the presence of the DT and moist tropical (MT) weather types in the winter in Los Angeles County were not associated with elevated levels of particulate matter < 2.5 *μ*m. Moreover, Santa Ana events tend to be windy, preventing the stagnation necessary for the build-up of winter pollution. Thus, it is unlikely pollution is playing a major role in contributing to the observed increases in human mortality.

## Conclusions

A strong relationship between winter heat and human health, especially among the elderly, has been uncovered in Los Angeles County, the first such study specifically examining the susceptibility of the population to heat in a mid-latitude U.S. city in the winter. We first utilize a synoptic approach, comparing air mass type to human mortality. Paying special attention to oppressive, DT and MT+ air, this methodology allows us to examine an entire weather situation rather than an evaluation of individual meteorological variables. Results provide some evidence supporting a winter heat-health link, particularly among the elderly and during a DT air mass. However, adjusting for large year-to-year variations in winter mortality unrelated to heat proved to be challenging, thus we also applied distributed lag nonlinear modeling to the dataset.

As the DLNM package offers the ability to control for variability at multiple scales, we were able to determine that relative risk of human mortality increases significantly under excessive winter heat, with the most robust relationships present for total mortality and those 65+ based on maximum and mean temperatures.

The discovery of heat-related mortality in the middle of the winter is an important finding, unique to Los Angeles County in the U.S. When excessive winter heat in Los Angeles occurs, it can cause substantial increases in winter mortality, and we believe more robust intervention techniques and increased awareness are necessary to help mitigate the impact of winter heat. Further, with the recognition of this problem, we encourage active collaboration among the various entities in Los Angeles County and City that are responsible for saving lives and increasing human well-being.

## Abbreviations

BUR: Burbank Bob Hope Airport
CDC: Centers for Disease Control and Prevention
DLNM: Distributed lag nonlinear modeling
DM: Dry moderate air mass
DP: Dry polar air mass
DT: Dry tropical air mass
EPA: Environmental Protection Agency
ICD: International Classification of Diseases
LAX: Los Angeles International Airport
MM: Moist moderate air mass
MP: Moist polar air mass
MT: Moist tropical air mass
MT+: Moist tropical plus air mass.
NCEI: National Centers for Environmental Information
NWS: National Oceanic and Atmospheric Administration/National Weather Service Offices
NZJ: Marine Corps Air Station in El Toro
RR: Relative risk of mortality
SSC: Spatial Synoptic Classification
TR: Transition air mass
